# Monitoring the Dynamics of Aquatic Vegetation in a Typical Shallow Lake Using the Water Bloom Index Algorithm—A Case Study in Bao’ an Lake in the Middle Reaches of the Yangtze River

**DOI:** 10.3390/plants13213090

**Published:** 2024-11-02

**Authors:** Shixing Song, Xiaodong Wu, Jianjun Hou, Shuang Peng, Xiaowen Lin, Xuguang Ge, Dongming Yan, Guiying Lin

**Affiliations:** 1 College of Urban and Environmental Sciences, Hubei Normal University, Huangshi 435002, China; 1261034849@stu.hbnu.edu.cn (S.S.); psh7840e@stu.hbnu.edu.cn (S.P.); lxw1039182719@stu.hbnu.edu.cn (X.L.); gxg76@hbnu.edu.cn (X.G.); lin20176@hbnu.edu.cn (G.L.); 2 Resource-Exhausted City Transformation and Development Research Center, Hubei Normal University, Huangshi 435002, China; 3 Huangshi Key Laboratory of Soil Pollution and Control, Hubei Normal University, Huangshi 435002, China; 4 College of Life Sciences, Hubei Normal University, Huangshi 435002, China; 5 Hubei Key Laboratory of Edible Wild Plants Conservation and Utilization, College of Life Sciences, Hubei Normal University, Huangshi 435002, China; 6 College of Geography and Planning, Chengdu University of Technology, Chengdu 610059, China; yandm936@126.com

**Keywords:** VBI algorithm, Bao’ an Lake, aquatic vegetation, coverage, remote sensing

## Abstract

Understanding changes in the distribution and coverage of aquatic vegetation (AV) is of great significance for the restoration of lake ecosystems. In this study, the vegetation and bloom indices (VBI) algorithm were used to interpret submerged aquatic vegetation (SAV), floating/emergent aquatic vegetation (FEAV), and algal bloom (AB). The dynamics of AV and their influencing factors in Bao’ an Lake, in the middle reaches of the Yangtze River in China, were studied from 2000 to 2023. The results showed that (1) the VBI algorithm can accurately distinguish AV and AB of different life forms with an overall accuracy of 93% and a kappa coefficient of 0.86. (2) Macrophyte coverage decreases. AV grew vigorously in spring, and SAV was the dominant type within it, whereas AV coverage was low in summer, and SAV had no summer species for a long time. In 2000, the coverage of AV was the highest, reaching 64.5%, but a gradual decrease that followed in the coming years finally led to a coverage percentage of less than 5% by 2023. (3) The correlation between SAV coverage and total phosphorus (*p* < 0.01), total nitrogen (*p* < 0.05), and water depth/transparency (*p* < 0.05) in Bao’ an Lake were 0.23, 0.28, and 0.32, respectively. (4) The SAV species experienced three stages: richness (before 2003), monotonicity (2004–2020), and final disappearance (2021–present). This study shows that the coverage of AV in Bao’ an Lake is too low and the number of SAV species is one (2010–now). Therefore, it is necessary to implement measures to improve vegetation coverage and diversity.

## 1. Introduction

Aquatic vegetation (AV) is a primary producer in lake ecosystems [1,2,3]. AV stabilizes sediments, reduces the release of nutrients [4], effectively slows down water flow, and provides food and habitat for benthic animals [5]. However, excessive AV growth may lead to changes in the ecological structure of shallow lakes, changing them from a clear-water state dominated by submerged aquatic vegetation (SAV) to a turbid-water state dominated by floating AV (FAV) and algae [6]. If excessive AV cannot be harvested or utilized in time, plant debris decomposes, releasing large amounts of nitrogen (N) and phosphorus (P) into the lake [7]. Moreover, the AV composition of lakes affects their aquatic environments. SAV promotes the transformation of lakes from a turbid-water state dominated by algae to a clear-water state dominated by macrophytes. By contrast, floating/emergent aquatic vegetation (FEAV) may cause lakes to become dominated by turbid algae [8]. Therefore, it is clear that monitoring the dynamics of changes and the distribution of AV forms is critical to the management and protection of lakes.

Traditional AV investigations of AV mostly adopt field investigations, which can accurately determine the species and their distribution to a certain extent. However, field investigations require considerable time and manpower and have certain limitations in the large-scale and long-term dynamic monitoring of AV [8,9]. Remote sensing (RS) technology is faster and more efficient for interpreting the distribution of AV in lakes and has gradually developed as the main method. Currently, RS is widely used for the spatial and temporal monitoring of AV in shallow and large lakes, such as Poyang Lake [10], Taihu Lake [8], Woods Lake, Michigan Lake, Lake Ontario, and Superior Lake [11]. The water transparency of these lakes was high, the impact of the RS information extraction was small, and the detection accuracy was high. Many studies have been conducted on AV classification. In RS interpretation methods for aquatic vegetation, the degree of manual participation can be classified as supervised Learning and unsupervised Learning classification [12]. Supervised Learning requires training samples based on human experience, and it is necessary to select an appropriate classifier based on the complexity and precision of classification. Commonly used classification methods include parallelepiped, minimum distance, Mahalanobis distance, maximum likelihood, random forest, SPA, and decision tree classifications [13,14,15]. Unsupervised Learning does not require prior knowledge and allows machines to learn and classify based on the ground spectral characteristics. Commonly used classification methods include convolutional neural networks, ISODATA classification, k-means classification algorithms, and deep learning (DL) methods [16,17]. Researchers have also developed automatic SAV classification algorithms [13]. However, the random forest and deep learning methods require sufficient samples and a longer time for simulation training [18]. Decision trees require complex algorithms (such as the CART algorithm) for construction, which can be extremely complicated. In 2023, Luo proposed a three-step classification algorithm based on Landsat satellite images and the vegetation and bloom indices (VBI) algorithm. The VBI algorithm is easy to operate, with a total accuracy of 84.49% [19]. It can efficiently identify SAV, FEAV, and algal bloom (AB), and it can be applied to interpret the interannual variation in lake vegetation.

Bao’ an Lake is a shallow lake located in the middle reaches of the Yangtze River. In recent years, the coverage of SAV in Bao’ an Lake has decreased, the number of species has been greatly reduced, and eutrophication has become an increasingly serious issue. For ecological restoration, the water quality index threshold should be considered along with the effects of positive feedback, functional characteristics, and species diversity [20]. At present, research on AV change in Bao’ an Lake mainly focuses on single or scattered years by field investigation [13,21,22,23]. There are few large-scale studies on the monitoring of AV changes, and there is no use of RS to monitor the distribution of AV in Bao’ an Lake. Moreover, the growth of AV is affected by many complex factors such as water level, fishery production, and sediment [13,14,22]. Therefore, it is of great significance for the ecological protection and restoration of Bao’ an Lake to interpret AV using RS and to analyze the relationship between AV distribution and environmental factors. In this study, the VBI algorithm was used to interpret Landsat images. Combined with field investigations, the seasonal and interannual changes in aquatic vegetation cover in Bao’ an Lake and its impact on the lake system were revealed, providing a reference for the restoration of AV in Bao’ an Lake.

## 2. Materials and Methods

### 2.1. Study Area

**Figure 1:**
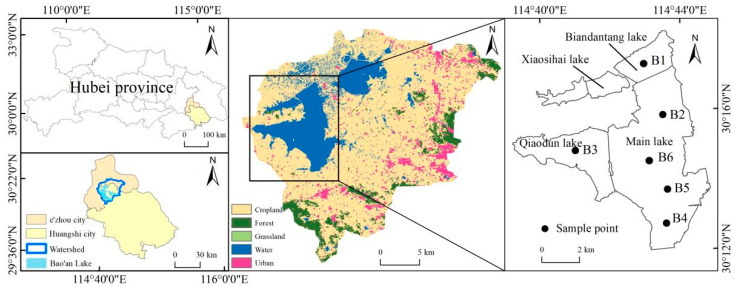
Study area.

Bao’ an Lake (30°12′0″–30°18′0″ N, 114°30′0″–114°45′55″ E) is located in Hubei Province, China, spanning Ezhou and Huangshi (Figure 1). The Bao’ an Lake area has a northern subtropical monsoon climate, with an average annual temperature of 16.5 °C. The average water depth of Bao’ an Lake is 1.5–2.5 m, and the existing lake area is 39.3 km^2^. The corresponding volume is 170 million m^3^ [20]. It can be divided into four lake areas: the main lake, Biandantang Lake, Xiaosihai Lake, and Qiaodun Lake. Lake water flows from south to north, and the water mainly originates from surface runoff. Its inflowing rivers include the Huandiqiao, Bao’ an Donggang, and Bao’ an Xigang Rivers in the south, followed by the Hejinggang River in the northeast (interlinked with Sanshan Lake). Water from Bao’ an Lake flows into the Changgang River from the Donggoumen Outlet, which is the drainage channel for Liangzi Lake, and it flows into the Yangtze River through the Fankou Gate.

### 2.2. Data Source

#### 2.2.1. Water Sampling and Quality Assessment

Six sampling points were established based on the area and morphology of Bao’ an Lake. A field survey of Bao’ an Lake was conducted every quarter from 2018 to 2023 (Figure 1). A mobile phone GPS toolbox (version 2.8.5.1) was used to record the coordinates of each sampling point. The Secchi disk depth (SD) was measured using a Secchi disk with a diameter of 30 cm, and the water depth was measured using a water depth meter. The organic glass water sampler was used to collect 500 mL water samples at 0.5 m underwater at each sampling point and put them into polyethylene bottles. The samples were placed in an incubator and sent to the laboratory for analysis. Total nitrogen (TN) and total phosphorus (TP) were determined using potassium persulfate oxidation-ultraviolet spectrophotometry (Shimadzu UV–visible spectrophotometer UV-2700i, Shimadzu Corporation, Kyoto, Japan) and potassium persulfate digestion (BXM-30R vertical pressure steam sterilizer, Shanghai Boxun Medical Biological Instrument Corp, Shanghai, China), respectively. Chlorophyll a was determined by 90% acetone extraction. Ammonia nitrogen (NH_3_-N) was determined using sodium reagent spectrophotometry [24].

#### 2.2.2. Satellite Data

The images used in this study were selected from Landsat satellite data. Among these, the TM and OLI data constituted the main part of the data in this study. Because of the influence of the satellite launch time and in-orbit date, image data before 2012 were obtained from Lantsat 4–5 TM, and image data after 2012 were obtained from Landsat 8 OLI_TIRS. In 2012, the repaired ETM + data were selected to supplement the data sequences. We selected April–May as the image data for spring, and July–August as the image data for summer. In each season, an image without cloud cover, external environmental interference, or sunny weather was selected as the research object, and the image data were derived from “http://www.gscloud.cn/” (accessed on 26 March 2024) and the Earth Explorer website.

Owing to the satellite revisit cycle and image quality, only the adjacent months were selected for analysis. Surface reflectance data were obtained after radiation and atmospheric correction using ENVI5.3.

### 2.3. Research Methods

#### 2.3.1. Classification Model Based on VBI Algorithm

The VBI algorithm is a three-step classification method based on Landsat images, that is, the aquatic vegetation index (AVI) derived from spike cap transformation. This index can be used to distinguish between AV and non-AV. The normalized difference vegetation index (NDVI) was used to distinguish between FEAV and SAV. The floating algae index (FAI) was used to distinguish ABand open water (OW) in non-AV [19]. After radiation and atmospheric corrections, each band of the image data obtained surface reflectance data. The vegetation sensitivity index was calculated by substituting the surface reflectance data of different bands into the equation. Based on the VBI classification algorithm, a decision tree (DT) was constructed using ENVI 5.3, and the vegetation sensitivity index (VSI) was applied to the DT to obtain the spatial distributions of SAV, FEAV, and AB.

#### 2.3.2. Vegetation Sensitivity Index

(1) Humidity-based AVI

In the three-dimensional space composed of brightness, greenness, and humidity, Luo et al. in 2023 found that humidity performs well in identifying AV and non-AV and developed the AVI. The index can better distinguish AV and non-AV [19]. The equation is as follows:

(1) $$AVI=-\sum_{i=1}^{6}k\left(\lambda_{i}\right)R\left(\lambda_{i}\right)$$

where the humidity of the band is i, which is related only to the sensor [19] and is the surface reflectance of the ith band.

(2) Floating algae index (FAI)

Hu et al. in 2009 [25] proposed the FAI. It was developed based on a linear baseline algorithm and relies on three bands: red, near-infrared (NIR), and short-wave infrared (SWIR). It can effectively extract information on cyanobacterial blooms in lakes and also eliminate the influence of thin atmospheric clouds. Moreover, it has been successfully applied to the extraction of AB from lakes. In this study, the FAI was used to distinguish between AB and OW in the non-AV range. The equations used are as follows:

(2) $$FAI=R_{NIR}-R_{NIR}^{\prime }$$

(3) $$R_{NIR}^{\prime }=R_{NIR}+\left[R_{SWIR1}-R_{Red}\right]*\left[\frac{\lambda_{NIR}-\lambda_{Red}}{\lambda_{SWIR1}-\lambda_{Red}}\right]$$

$R_{NIR}$ refers to the near-infrared band surface reflectance, $R_{NIR}^{\prime }$ refers to the baseline reflectance obtained by linear interpolation between the red and NIR bands, and it is the NIR wavelength.

(3) Normalized difference vegetation index (NDVI)

The NDVI is an important parameter that reflects surface vegetation coverage, and its value can be used to distinguish between macrophytes and water bodies in lakes. The value of the NDVI is limited to [−1, 1]. When NDVI is less than 0.1, there is almost no vegetation, while one close to 1 indicates that vegetation grows vigorously, and NDVI values of deserts, OW, and other terrains are very low or negative. The equations used are as follows:

(4) $$NDVI=\left(NIR-R\right)/\left(NIR+R\right)$$

NIR is the value of the near-infrared band and R is the value of the red band.

(4) Normalized Difference Water Index (NDWI)

In 1996, McFeeters proposed an NDWI based on the green and NIR bands [26]. In this study, water was extracted, and the average AVI value of the water was calculated. The equation is as follows:

(5) $$NDWI=\left(p\left(Green\right)-p\left(NIR\right)\right)/\left(p\left(Green\right)+p\left(NIR\right)\right)$$

where p (Green) and p (NIR) refer to the green and NIR bands, respectively.

#### 2.3.3. Classification Steps

**Figure 2:**
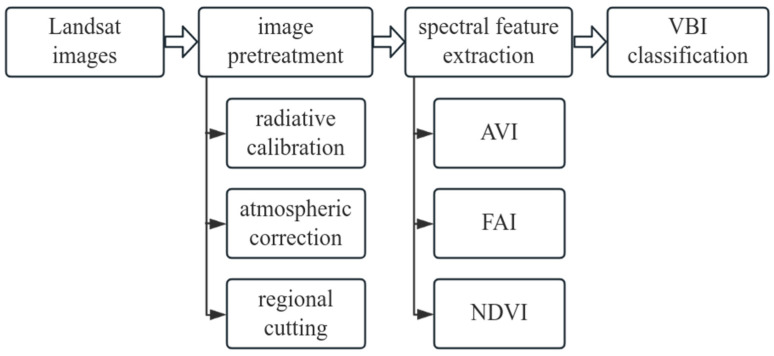
VBI algorithm classification steps.

ENVI 5.3 was used to analyze the image, and a DT was used to classify the pixels (Figure 2). The established model based on the VBI algorithm was applied to Landsat images of Bao’ an Lake in the spring and summer from 2000 to 2023, and spatial distribution maps of SAV, FEAV, and AB in Bao’ an Lake were obtained.

### 2.4. Data Analysis

The RS data of this study were processed using ArcGIS 10.6 and ENVI 5.3. Excel 2016 was used to sort the data. The relationships between the AV coverage and water depth/transparency (Z_M_/Z_SD_), TP, and TN were linearly fitted using IBM SPSS Statistics 27. *p* < 0.05 was significant, and *p* < 0.01 was extremely significant. The study area was drawn using ArcGIS 10.6, and other charts were drawn using Origin 2023.

## 3. Results

### 3.1. Accuracy Verification

The kappa coefficient is often used to evaluate classification accuracy. The closer the value is to one, the higher the classification accuracy. The overall accuracy was proportional to the number of correctly classified samples. Therefore, we used the kappa coefficient and overall accuracy to verify the accuracy of the interpreted images (Table 1). After verification, the overall accuracy of satellite image interpretation result was 93% and the kappa coefficient was found to be 0.87.

### 3.2. Interannual Variation of Aquatic Vegetation Coverage in Bao’ an Lake from 2000 to 2023

According to the field survey in 2019, there are eight types of aquatic vegetation in Bao’ an Lake, among which seven types of FEAV are *Typha orientalis Presl*, *Canna indica* L., *Nelumbo nucifera*, *Phragmites communis (Cav.) Trin. ex Steud. Trapa bispinosa Roxb.*, *Nymphaea* L., and *Lemna minor* L. The number of SAV types has decreased from eight in 1997 to only one now, leaving *Potamogeton crispus* L.

**Figure 3:**
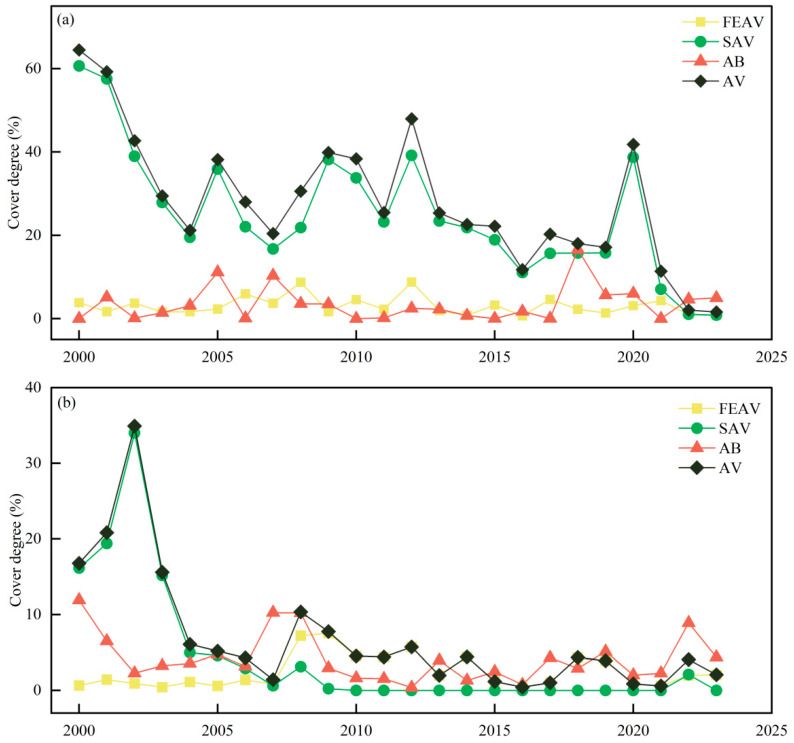
Different life forms of AV and AB coverage in spring (**a**) and summer (**b**) from 2000 to 2023.

From 2000 to 2023, the AV coverage in Bao’ an Lake showed a downward trend in spring (Figure 3). The observed change in AV in Bao’ an Lake in spring was the same as that of SAV coverage. The coverage of SAV in the lake was 60.6% in 2000 and then fluctuated, but overall there is a downward trend, less than 5% by 2023. The interannual variation in FEAV coverage was small, and the coverage varied between 1 and 9%. When SAV coverage reached the peak of each cycle, FEAV coverage was low, indicating a tradeoff between the aforementioned vegetation forms. The coverage of AB was low (0–2%). There was a large number of AB in the lake only in 2007 and 2018, with coverage of 10.3% and 16.5%, respectively.

When compared with the spring period, AV coverage decreased significantly in Bao’ an Lake during the summer period of the same time interval (2000–2023) (Figure 3). AV coverage experienced two stages: one before 2004, when the coverage was large and dominated by SAV, and the other after 2004, when the coverage was small and dominated by SAV. The coverage of SAV changed significantly in the summer, reaching a maximum coverage (34%) in 2002, and then it decreased rapidly until SAV disappeared in 2010. The coverage of FEAV did not change much compared with that in spring, and it increased first and then decreased as a whole. The coverage was low (1 ± 0.5%) from 2001 to 2007. From 2008 to 2012, it remained at a relatively high level (6 ± 1%); from 2013 to 2023, the coverage showed a decreasing trend, with a minimum of only 0.42%. The coverage of AB increased compared with that in the spring period, and the fluctuation was more obvious overall.

### 3.3. Spatial Distribution of AV in Bao’ an Lake

**Figure 4:**
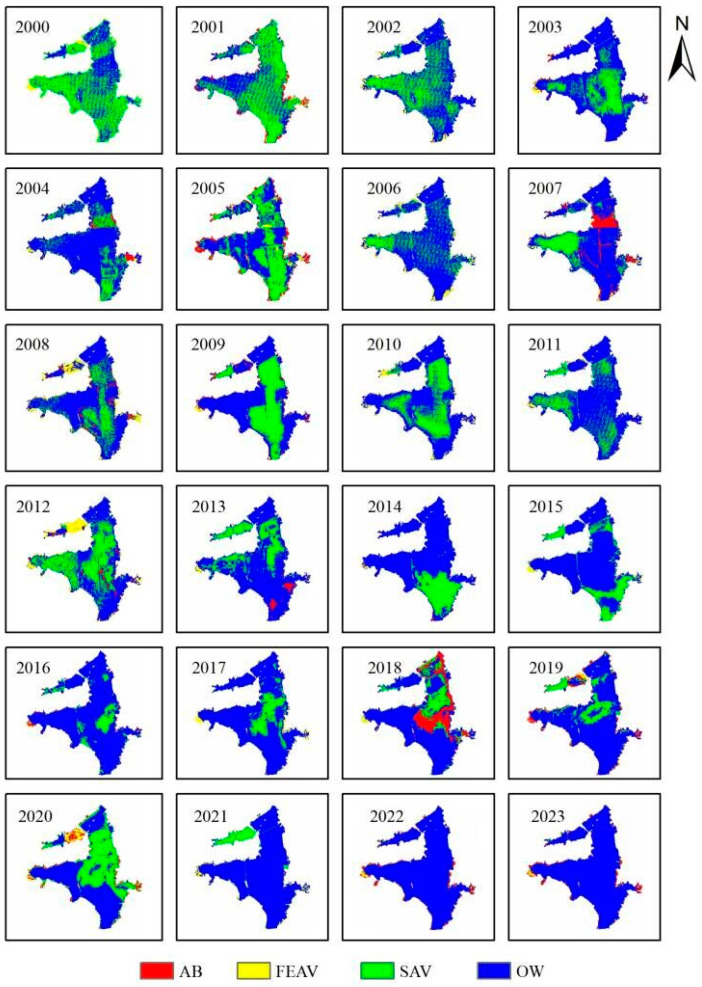
Distribution of AV in Bao’ an Lake in spring from 2000 to 2023.

Between 2003 and 2023, SAV distribution in Bao’ an Lake showed a downward trend and clear interannual variation during spring (Figure 4). Prior to 2003, SAV was distributed throughout Bao’ an Lake. After 2003, spatial heterogeneity was observed as SAV diminished and was located mainly in the main lake and Qiaodun Lake. The SAV distribution in Xiaosihai and Biandantang Lakes was more dispersed, and the coverage showed no clear interannual variation. The distribution of FEAV (during spring) was relatively scattered in the shallow waters along the shore of Bao’ an Lake. For several years, FEAV has been widely distributed in Xiaosihai Lake.

**Figure 5:**
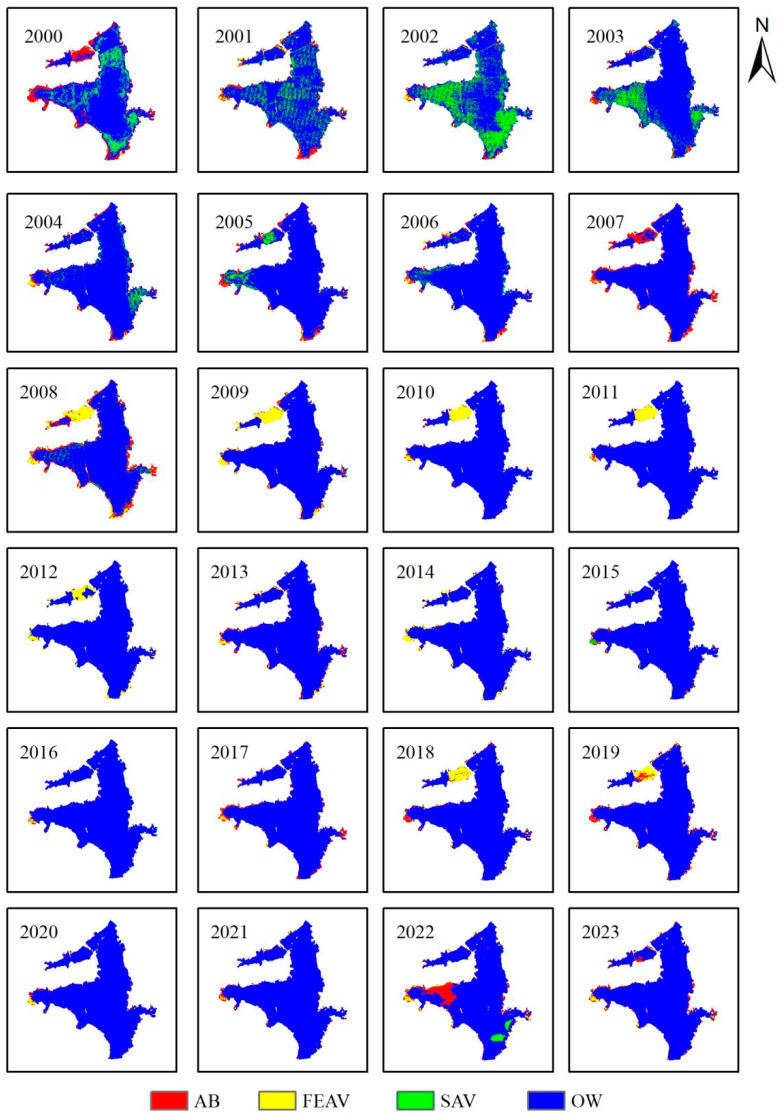
Distribution of AV in Bao’ an Lake in summer from 2000 to 2023.

The coverage of SAV in Bao’ an Lake changed significantly during the summer (Figure 5). From 2001 to 2003, the coverage of SAV on the lake surface decreased significantly compared to in spring, and the distribution was more dispersed. In 2003, SAV began to decline significantly and completely disappeared in 2010. Until 2023, there was no SAV in Bao’ an Lake. In summer, the distribution of FEAV was found to be scattered in the shallows along the shore, and the spatial heterogeneity was small. However, the inversion results showed that the distribution of FEAV in Xiaosihai Lake increased significantly. In 2008–2012, 2018, and 2019, FEAV was most concentrated in Xiaosihai Lake, whereas in other years, FEAV grew only in Xiaosihai Lake and the shoals along Bao’ an Lake.

### 3.4. Relationship Between AV and Environmental Factors

**Figure 6:**
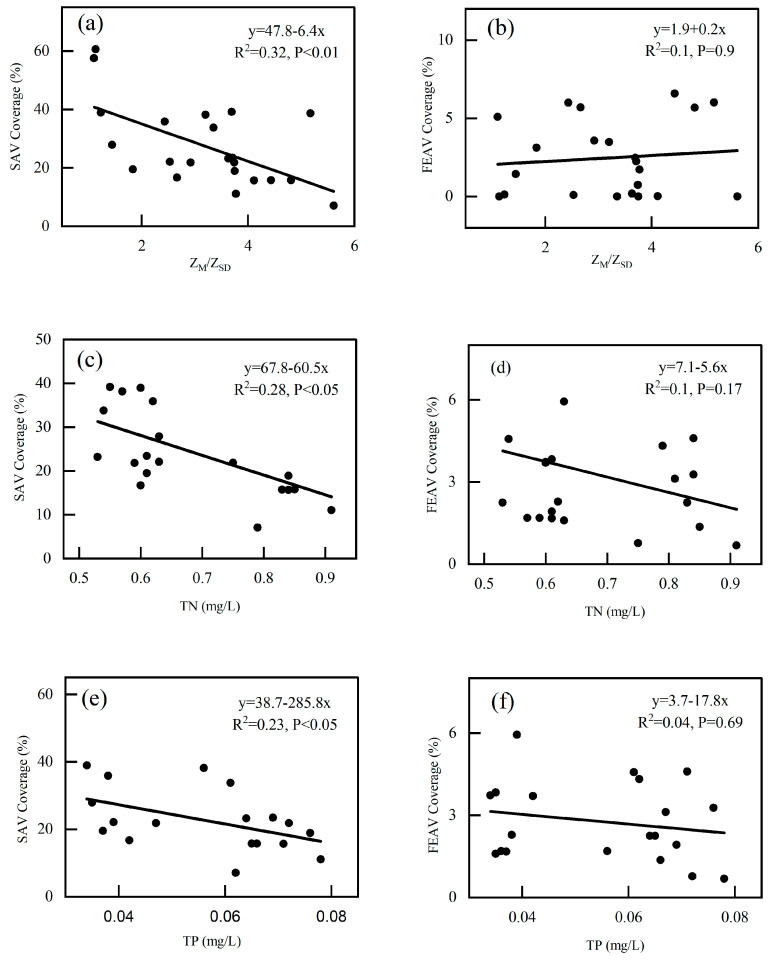
The correlation between Z_M_/Z_SD_, TP, TN, and AV coverage. Figure (**a**,**b**) show the linear correlation between SAV, FEAV and Z_M_/Z_SD_, respectively. Figure (**c**,**d**) show the linear correlation between SAV, FEAV and TN, respectively. Figure (**e**,**f**) show the linear correlation between SAV, FEAV and TP, respectively.

The AV of Bao’ an Lake is more abundant in spring; therefore, this study aimed to interpret the distribution and interannual variation of AV during spring. There was no significant correlation between the water level of Bao’ an Lake and the SAV coverage (*p* = 0.069). However, Z_M_/Z_SD_ was significantly negatively correlated with SAV coverage (R^2^ = 0.32, *p* < 0.01) (Figure 6). When Z_M_/Z_SD_ was greater than 5.17, SAV disappeared. However, there was no significant correlation between FEAV and water level (*p* = 0.48) or Z_M_/Z_SD_ (*p* = 0.9).

**Figure 7:**
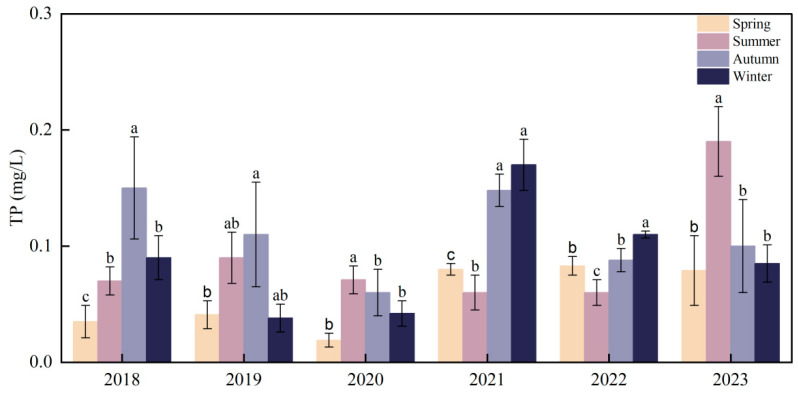
TP in Bao’ an Lake from 2018 to 2023. The error bar represents the standard deviation (SD), and different lowercase letters represent seasonal differences (*p* < 0.05).

From 2004 to 2023, the development of submerged vegetation in Bao’ an Lake was recorded only during spring, and there was almost no SAV growth in the other three seasons. In lake ecosystems, P is considered a limiting factor [27,28]. In this study, by comparing the water quality of the periods with or without SAV growth, it was found that when there was a large amount of SAV biomass, such as during the spring seasons of 2018–2020, the mean value of TP was 0.032 mg/L, which was significantly lower than that in the period without SAV growth, such as the summer, autumn, and winter of 2018–2020, and for the entire year 2021–2023, the mean value of TP was 0.085 mg/L (Figure 7). Simultaneously, SAV coverage in Bao’ an Lake was significantly correlated with TP (R^2^ = 0.23, *p* < 0.05) and TN (R^2^ = 0.16, *p* < 0.05), and with increasing TP levels, a significant reduction in SAV coverage was observed (Figure 7).

## 4. Discussion

### 4.1. The Impact Factors of AV in Bao’ an Lake

The FEAV in Bao’ an Lake is relatively scattered in the lake with a coverage of less than 10%, mainly concentrated in the shallow riverine areas. In some years, due to artificial planting, there is a large amount of FEAV growing in Xiaosihai Lake. The research results indicate that there is no significant correlation between the FEAV of Bao’ an Lake and various water quality indicators, and the change in coverage is not significant (s = 2.2). Therefore, in the subsequent discussion process, this study will only focus on the changes in SAV coverage

#### 4.1.1. Water Level and Z_M_/Z_SD_

**Figure 8:**
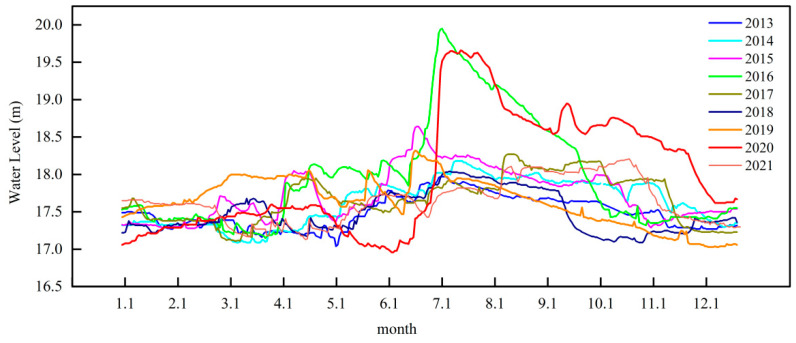
The water level change in Bao’ an Lake from 2013 to 2021.

The water level is an important factor in determining the distribution and structure of AV. This affects the germination, growth, and reproduction of AV [29,30,31]. The water level of Bao’ an Lake changed regularly from 2012 to 2021. The flood season occurs in mid-June each year, and the water level rises until the end of the flood season (late September to early October) and then gradually decreases (Figure 8). Bao’ an Lake is not fed by a river but is connected to Liangzi and Sanshan Lakes through the Donggou and Hejing Gates, respectively. The amount of water in the lake is controlled. The water level in the flood season fluctuates between 17.58 and 18.81 m, and the lowest water level appears in the spring (17.06–17.98 m) month. Seasonal and interannual variations in water-level fluctuations were relatively small. Emergent vegetation has strong adaptability to short-term or annual water-level fluctuations owing to its well-developed roots; therefore, water-level fluctuation mainly affects SAV. When the water level is too high and the SAV lacks light, its growth is blocked and the biomass is reduced. However, if the water level is too low, the SAV may be damaged by wave action or drought [30]. However, the results showed no significant correlation between SAV coverage and the water level in Bao’ an Lake (*p* = 0.09). This may be because the annual average water level of Bao’ an Lake does not change significantly (17.69 ± 0.16 m), and it seems that small fluctuations in water level cannot be causing the change in coverage directly. Therefore, the change in SAV coverage in Bao’ an Lake was affected by other factors. Bao’ an Lake experienced two extreme water-level fluctuations in 2016 and 2020. During the flood season, the average water level increased by more than 2 m, which may have been the cause of the significant disappearance of SAV in 2021. However, the high water level in 2016 did not lead to a similar phenomenon; therefore, the impact of water-level fluctuations on SAV growth and dispersion needs to be analyzed.

It can be seen from the above that the annual water level of Bao’ an Lake does not change significantly, and transparency can affect the underwater light intensity combined with the water level, which may be another factor in the change in SAV coverage in Bao’ an Lake. Studies have shown that when Z_M_/Z_SD_ exceeds 5.59, some AV does not grow [32]. The Z_M_/Z_SD_ ratio in Baoan Lake reached a maximum of 5.2 in 2020, which did not reach the level that could affect the growth of SAV. However, in 2020, when Z_M_/Z_SD_ reached 5.2, the highest water level during the flood season in Bao’ an Lake also reached 19.65 m. A high water level of above 18 m lasted until December. *Potamogeton crispus* (*P. crispus*) sprouted in October and grew during the winter. High water levels and Z_M_/Z_SD_ affected SAV growth in the following year. During a field investigation in the spring of 2021, there was no SAV growth in the main Bao’ an Lake, and unrooted *P. crispus* sprouts were floating. The average SD in summer was 35 cm, and Z_M_/Z_SD_ also reached 5.6, which was no longer suitable for SAV growth. In the same year, a similar situation occurred in Honghu Lake; however, in addition to *P. crispus*, there were other types of SAV in Honghu Lake, such as *Hydrilla verticillata*, *Ceratophyllum demersum* L., and so on. Therefore, this did not lead to the disappearance of all SAV in Honghu Lake.

#### 4.1.2. Fishery Production

The species and quantity of fish have significant effects on the growth and reproduction of AV and species changes. The main fish cultured in Bao’ an Lake are silver, bighead, and grass carp. Silver and bighead carp are filter-feeding fish that can resist microcystins and achieve rapid growth by feeding on toxic cyanobacteria. Cyanobacterial blooms can be reduced using non-classical biomanipulation. However, if the culture density is too low, the control effect on cyanobacterial blooms is reduced. However, high-density fish disturb the sediment, resulting in resuspension [33]. Therefore, the silver carp and bighead carp in Bao’ an Lake can avoid outbreaks of cyanobacterial blooms to a certain extent. The fishery culture of the Bao’ an Lake has undergone three stages. The first stage was the enclosure, blocking, and net cage breeding stage, which emerged after the construction of embankments and sluices in 1970. Large-scale stocking of grass carp leads to a large amount of AV being gnawed, affecting their growth, and the eutrophication of water bodies is caused by large amounts of fertilizer application. However, nutrient levels before 2002 were low and did not significantly inhibit SAV growth [22]. This also makes the RS interpretation results show that the coverage of AV was largest in the spring before 2002, and SAV was distributed in the summer before 2002. In the second stage, the seine was removed in 2010 and the fish in Bao’ an Lake were regularly fished. Although the stocking of silver carp and bighead carp does not cause significant damage to SAV, fishing activities can affect the growth of AV [22]. At this stage, *P. crispus* was the dominant population in Lake Bao’ an. Owing to its strong resistance to pollution [30], although SAV coverage fluctuates, it generally remains relatively stable. In 2018, the lake was completely closed for fishing, particularly for silver and bighead carp. Moderate fishing reduces the effect of fish fauna on aquatic plants. Therefore, in the spring of 2018–2020, AV coverage in Bao’ an Lake began to show an upward trend.

Fishery production affects AV coverage in Bao’ an Lake and AV species. The fish species cultured in Bao’ an Lake are mainly grass, silver, and bighead carp. In the early stages of fish farming, large amounts of grass carp [22], silver carp, and bighead carp were used while they were introduced into the lake to improve economic output. Grass carp have strong palatability to *Vallisneria natans* (*V. natans*). Therefore, with an increase in grass carp culture density, *V. natans* was consumed in large quantities. Subsequently, when the *V. natans* vegetation was reduced, the stocking amounts of silver and bighead carp increased. Silver and bighead carp can effectively reduce cyanobacterial blooms without directly affecting SAV [33]. Due to the change in the aquaculture mode in Bao’ an Lake, the diversity of AV decreased, as the number of submerged plant species (SAV) showed a severe reduction. In the 1980s, there were ten common SAV species (*Potamogeton wrightii*, *Potamogeton maackianus*, *P. crispus*, *Najas marina*, *Najas minor*, *Hvdrilia verticillata*, *V. natans*, *Ottelia alismoides*, *Ceratophylum demersum*, *Myriophyllum spicatum*), which decreased to seven species in the 1990s. Since 2000, SAV species have declined further, and *P. crispus* has become the only SAV species [22].

#### 4.1.3. Sediment Nutrients

Bao’ an Lake is located in the suburbs of Daye City adjacent to Liangzi Lake, a typical suburban lake. Under the influence of surrounding villages and aquaculture, large amounts of N and P accumulate in sediments. Although sediments help maintain the ecological balance of lake water properties, the re-release of N and P from sediments into the water body is a major cause of eutrophication [34]. The growth of algae is strictly dependent on the nutrient availability of water bodies, rather than the roots of aquatic plants that mainly obtain nutrients from sediments; therefore, it is more suitable for eutrophic water bodies. However, the main nutrient source for SAV growth is sediment. Water eutrophication inhibits the growth of SAV and promotes growth of algae [35]. Eutrophication affects the biomass and species by affecting the morphological complexity and plasticity of aquatic plants, ultimately affecting the entire ecosystem [20]. This chain reaction eventually reduces ecosystem resilience. Under normal circumstances, the growth of algae in areas where macrophytes grow in lakes is inhibited. When the amount of water flowing into the lake during the flood season increases, the waterbody becomes significantly disturbed. At the shallow water depth of the lake, the disturbance of wind on the sediment leads to an increase in the content of organic matter (OM) in the water layer, and a large number of algae grow, reducing underwater light, and thus inhibiting the growth of SAV. Simultaneously, the sediment originally fixed by SAV was resuspended, further increasing the suspended particulate OM in the water layer and making the water more turbid. The sediment pollution index of Bao’ an Lake indicated severe pollution (IV), primarily N and OM [36]. *P. crispus* has strong pollution resistance; therefore, it has become the dominant species in Bao’ an Lake [34]. However, TP in the overlying water had a significant impact on the AV. Studies have shown that in shallow lakes when TP exceeds 0.08–0.12 mg/L, the lake will change from a clear-water state to a turbid-water state [37]. The SAV coverage of Bao’ an Lake was also negatively correlated with TP (Figure 6). The SAV coverage decreased with increasing TP. The concentration of TP in Bao’ an Lake was 0.035–0.078 mg/L over the years, which did not exceed this threshold but was very close.

Studies have shown that when the wind speed of Taihu Lake exceeds 3.4 m/s, the rate of sediment resuspension is greater than that of the sedimentation rate [27]. Although there is currently no information on the critical wind speed for sediment resuspension in Bao’ an Lake at present; however, both Bao’ an Lake and Taihu Lake are shallow lakes. The average wind speed in Bao’ an Lake is 2.1 m/s, whereas the maximum wind speed is 10 m/s. Therefore, wind waves also promote the resuspension of sediment in the open water area of Bao’ an Lake to a certain extent. Moreover, many fish disturbances and fishing activities in the seine culture stage during the fishery production process are important causes of sediment resuspension.

### 4.2. Implications for AV Restoration in Bao’ an Lake

The number of AV species is one of the factors affecting the stability of lake ecosystems. If the number of species is too low, the stability of the ecosystem is reduced and the ability to resist external interference is weak [28]. In recent years, SAV in Bao’ an Lake has disappeared due to human activities and natural factors. Appropriate AV should be selected for supplementary planting, such as eutrophic *P. crispus* and *V. natans*, to increase species richness. Moreover, serious pollution of Bao’ an Lake sediment should be limited, and measures should be taken to control the release of sediments. Scientifically, regulation of the water level is needed to ensure that it remains low in winter and spring, thus facilitating the germination of AV species, optimizing the fishery structure of Bao’ an Lake, and controlling fish density and biomass.

## 5. Conclusions

Based on Landsat images, this study interpreted the distribution and coverage of AV in Bao’ an Lake during spring and summer from 2000 to 2023 using the VBI method. This study analyzed the relationship between water level, transparency, fishery production, sediment, and AV coverage in Bao’ an Lake during spring.

(1) It was found that the VBI algorithm can be applied to the classification of AV in Bao’ an Lake because the classification accuracies for FEAV, AB, and SAV can reach 93%.
(2) The AV coverage in Bao’ an Lake in spring was dominated by SAV, and the overall trend declined. In summer, the AV coverage is dominated by FEAV, and summer SAV species were missing.
(3) The coverage of SAV in Bao’ an Lake is negatively correlated with TP and TN and seems to be affected by the combined effect of water level and transparency. When the ratio Z_M_/Z_SD_ exceeded 5.2, the SAV growth was inhibited.
(4) SAV species richness experienced three phases: the species-rich first period, the period during which only one species existed, and finally all species disappeared. SAV disappeared under the influence of a high-water level and low transparency.
(5) The sediments of Bao’ an Lake are mainly resuspended under the influence of fish disturbances and wind waves, which aggravate the eutrophication of the water body and affect aquatic plants. In the early stages of purse seine aquaculture in Bao’ an Lake, fish disturbance had the strongest effect on sediment resuspension. To date, the impact of aquaculture on Bao’ an Lake has been minimized.

At present, the AV coverage of Bao’ an Lake is low, there is no SAV growth, and sediment pollution is serious. Therefore, measures should be taken to recover the SAV communities and also prevent sediment resuspension.

## Figures and Tables

**Table 1:** Classification results of AV.

	FEAV	SAV	AB	OW
FEAV	10	0	0	0
SAV	88	775	17	0
AB	0	0	160	0
OW	11	88	6	2036
Kappa Index	0.87			
Overall accuracy	93.42%			

## Data Availability

The datasets generated and/or analyzed during the current study are available from the corresponding author on request.
